# Support needs of young adults on antiretroviral therapy in Capricorn District, Limpopo province

**DOI:** 10.4102/hsag.v28i0.2125

**Published:** 2023-07-31

**Authors:** Tumisho Mokwele, Dorricah Peu, Moeta Mabitja

**Affiliations:** 1 Department of Nursing Science, Faculty of Health Sciences, University of Pretoria, Pretoria, South Africa

**Keywords:** adherence, antiretroviral therapy, needs, support, young adults

## Abstract

**Background:**

Human immunodeficiency virus (HIV) has been a major focus of our healthcare system. Over time, treatment policies have been changed to improve the lives of people living with HIV, which led to the introduction of the universal test and treat policy. People living with HIV require support to ensure that they are not lost to follow-up and that they adhere to antiretroviral therapy (ART).

**Aim:**

This study aimed to explore and describe the support needs of young adults on ART in Capricorn District, Limpopo province.

**Setting:**

The study was conducted at a public clinic in Capricorn District, Limpopo province.

**Methods:**

A qualitative, explorative, descriptive research and a convenience sampling method was used. Data were collected face to face using unstructured interviews from 20 young adults, who were selected using inclusion and exclusion criteria. Data analysis were done using Teschs’ method of analysis.

**Results:**

This study indicated that the family played a major role in ensuring that their loved one adhered to a medical regimen and attended their follow-up care. The importance of continuous counselling and education by healthcare workers (HCWs) was of importance as they encourage adherence.

**Conclusion:**

Support is an important factor required to ensure that young adults on ART remain in care and attend follow-up care.

**Contribution:**

The study informs the family and HCWs about the support needs of young adults on ART, which when met, can contribute to a positive outcome of treatment adherence.

## Introduction

Support for people living with human immunodeficiency virus (HIV) on antiretroviral therapy (ART) is important to ensure positive outcomes (Moosa et al. 2019:10). The National Department of Health (NDoH 2016) issued a communication regarding the implementation of the Universal test and treat (UTT) policy which advised healthcare workers (HCWs) to initiate treatment for people living with HIV after knowing their status. This, however, like any new policy, had its challenges as it meant that asymptomatic people would have to be on ART (Onoya et al. 2021:9) which increased the risk of people without support not adhering to medication.

According to the Joint United Nations Programme on HIV and acquired immunodeficiency syndrome (AIDS) data (2020) epidemiological estimates, there were 38 million people globally who were living with HIV in 2019. Of those infected, South Africa accounted for 7.5 million infections (UNAIDS 2020). The Limpopo province accounted for 51 5091 infections according to the Treatment Action Campaign Report (Low & MacDonell 2019). Low and MacDonell (2019) further indicated that Limpopo had 57.24% of people living with HIV, whose viral load is below 1000 c/mL, which meant that the province has 42.76% people who were not suppressing well to ART. Low and Macdowell’s (2019) findings indicated that Limpopo province has not yet reached its goal, in line with the 90-90-90 strategy.

South Africa has one of the biggest rollout programmes for ART in the world and young adults accounted for 71% of those on ART (Centre for Disease Control and Prevention 2019). In a country where 34.5% of young people aged 15–34 years are unemployed, it is difficult for them to support themselves and their families financially (Statistics South Africa 2022:15). Therefore, the issue of support becomes a major aspect in ensuring that they remain in care. The support required depends on their needs, be it psychological, emotional, financial or any other form.

Mbonye et al. (2016:37) observed that although people may be in ART programme the challenges they face should not be ignored as they may be lost to follow-up if not attended to. This was also supported by Horvath et al. (2019:11), as they found that young people had interpersonal, social, structural and cultural challenges, which will have an impact on continuing with HIV care. With the introduction of UTT, Nhassengo et al. (2018:11) found that there are different factors, which can become barriers to ART adherence, these factors included feeling healthy, non-acceptance of the HIV status, poor knowledge about ART, fear of ART side effects and fear of disclosure because of fear of stigmatisation became barriers to HIV care. Previous literature related to UTT focuses more about its importance, failing to look into the support for young adults that may be needed to ensure that young adults remain in care and are adhering to ART (Koenig et al. 2017:12; Lilian et al. 2020; Pilcher et al. 2017:8). Therefore, support can only be offered if the individual needs are known.

### Aim

This study aimed to explore and describe the support needs of young adults on ART in Capricorn District, Limpopo province.

## Research methods and design

### Study design

A qualitative, explorative, descriptive and contextual method was used for this research as it allows the phenomenon to be investigated in-depth (Polit & Beck 2017:741). The support needs of young adults on ART in Capricorn District, Limpopo province were explored and described.

### Setting

The research was conducted at a public clinic in Capricorn District, which in 2017, had 105 208 people living with HIV in care (Molapo & Massyn 2019:204). The clinic was selected as it only provides HIV testing and counselling services, and it also offers treatment for those who have tested positive for HIV. It serves people living with HIV from both formal and informal settlements. The District Development Model (2020) indicated that Capricorn District had a population of 1 372 355, where 46% were adults aged 20–59 years of age. The area where the research was conducted had 24-hour health facilities and a district hospital.

### Study population and sampling strategy

The population sample included young adults aged 21–34 years (male or female) who were diagnosed on or after 01 September 2016 as they were of consenting age to participate in research. It was required that they had to be on ART from 01 September 2016 with the commencement of the UTT and treat protocol. They had to be collecting treatment at the selected clinic in Capricorn District, Limpopo province. Participants were recruited from January 2020 to February 2020.

A convenience sampling method was used in order to select participants, with the help of the Operational Manager of the Clinic. This method was used as the participants were readily available, as they were coming for follow-up care at the clinic (Polit & Beck 2017:724). The researcher was granted permission by the Hospital Chief Executive Officer who then introduced the researcher to the operational manager. The researcher then made an appointment with the operational manager. On the set date, the researcher went to Capricorn District at the selected public clinic to provide information to patients who had been on ART under UTT at the clinic. The clinic offered services to people living with HIV only which meant that information sessions were done in the morning before patients could commence with their consultation. The researcher introduced herself to the patients who were at the clinic for follow-up care. Leaflets were given to the patients and were read to them in either English or Sepedi to ensure that those who were unable to read were not excluded. The leaflets contained the research aim and objectives of the study. Participants were given 2 to 3 weeks to decide whether or not they would participate in the research. Contact details of the researcher was given to the participants who wanted to take part in the study. The participants were advised to call or send a Please Call Me to the researcher, who thereafter called them to confirm the date and the place for the interviews.

### Data collection

A pilot study refers to a small-scale version of the study conducted to prepare for the major study. A pilot study, as referred to by Polit and Beck (2017:739), is designed to assess the feasibility of the study. In this study, it was performed to assess whether the methodology, sampling and analysis used in the main study were sufficient and applicable to answer the research questions (De Vos et al. 2011:206).

The researcher conducted face-to-face pilot study with two participants using unstructured individual interviews. On the day the interviews were arranged with participants, the researcher re-read the leaflet information, the procedure was explained and the consent form was signed. The main question was posed and different skills of communication were used such as probing, paraphrasing and clarification to prompt more information from the participants. During the pilot study, the researcher did not have a designated area to conduct interviews as the office was also used by the dietician. The operational manager assisted by allowing the researcher to use her office during subsequent interviews to reduce distractions. The findings of the pilot study were not included in the main findings as they were to assess the feasibility of the study. The pilot study was a success and therefore no changes were made.

Data were collected from 20 young adults and data saturation was reached, using unstructured interviews after consent was granted. The face-to-face interviews were carried out in English and Sepedi as it is well understood in this setting. One interview was collected from the home of the participants as she could not come to the clinic for our appointment, one near their workplace and 18 were collected from the clinic in the operational manager’s office. An audio recorder was used, and the participants signed a consent form before the recording commenced. The interviews were labelled Participant 1 to Participant 20. The following key question was asked of the participants:

‘What are your support needs since you started taking antiretroviral therapy?’

The question was followed up by various communication strategies such as probing, clarification and paraphrasing. An audio recorder that participants consented to was used, which enabled the researcher to transcribe interviews without leaving out important information. The recorded interviews were translated to English by the researcher as she is Sepedi speaking, and therefore the meaning was not distorted.

The recorded data were collected over a maximum period of 30 min. After it was collected, all individual participants were thanked.

### Data analysis

Data were analysed using Tesch’s eight steps (Creswell 2014:198) in a systematic process, which was performed according to the following sequence:

- The researcher read through the transcripts getting to have logic of the data gathered.
- The researcher read the participant’s interviews to understand what it was about.
- After reading the participant’s interviews a list with similar themes was made and put together making sure to have them arranged into major themes and unique themes.
- The themes were abbreviated as codes and written on the relevant part of the text in order to organise data and to see whether new categories and codes would emerge.
- The researcher gave codes to descriptive words that were turned into categories, which were reduced by grouping similar categories to show interrelationships.
- The researcher made a final decision on the abbreviations for each category and alphabets were assigned to each category to ensure that there was no duplication.
- Similar categories were grouped in one place focusing on the content of every category and a preliminary analysis was carried out.

Transcribed data by the transcriptionist was then given to an independent coder who has experience in qualitative data analysis. After the independent coder completed the data analysis, findings were agreed upon.

### Trustworthiness

Trustworthiness was ensured through credibility, confirmability and dependability. Credibility was ensured through prolonged engagement and member check. Prolonged engagement was attained through spending sufficient time with participants, in order build trust and rapport between the researcher and the participants. The researcher collected data from February 2020 to March 2020, a prolonged period, until data saturation was reached and until no new information was generated.

When data were collected the researcher was able to summarise and emphasise what was said by the participants to determine accuracy during the interview process to ensure member checking. The researcher gave the audio recorder and field notes used for interviews to an independent coder to be analysed to ensure dependability. Interviews were recorded by the researcher to ensure that transcribing of data were performed verbatim by the transcriptionist after collecting data. During the analysis of data, independent coder’s services were utilised to ensure that the study is not biased and that it fully represents the views of the participants. Data were given to an independent coder after it was transcribed. The findings were discussed by the researcher and the independent coder, and the themes and categories were agreed upon. The given report reflected the views of the participants.

## Results

The participants who were interviewed were 16 females (80%) and 4 males (20%) of which 7 (35%) were employed and 13 (65%) were unemployed. The demographics for the participants are indicated in Table 1.

**TABLE 1:** Demographic information of participants.

Criterion	Characteristic	Frequency	
		*n*	%
Gender	Female (F)	16	80
	Male (M)	4	20
Employment status	Employed	7	35
	Unemployed	13	65

During data analysis, three themes and five categories emerged. These included (1) family and friends support, (2) facility support and (3) status disclosure. Each of these themes is supported by participants’ quotes. The themes that emerged are presented in Table 2.

**TABLE 2:** Themes and categories.

Theme	Categories
Family and friends’ support	1.1 Motivation and encouragement 1.2 Financial support
Facility support	2.1 Counselling sessions 2.2 Education about treatment
Status disclosure	3.1 Duration and disclosure to family and friends

### Theme 1: Family and friends’ support

The theme ‘family and friends’ support’ emerged as one of the main support needs of young adults initiated on ART. The support from family and friends were in the form of motivation and encouragement and financial support.

#### Category 1.1: Motivation and encouragement

Words of motivation and frequent encouragement from family and friends were also cited as beneficial for participants. Participants mentioned that the advice on what to do at certain times and the consequences of not taking the prescribed medications compelled them to adhere to ART.

In terms of motivation and encouragement, one participant mentioned:

‘She [*her mother*] instead supported me, she sat me down and she told me that this is how a lot of people live, that I should stay away from alcohol and street things and drink my medication then I will be all right.’ (P8, M, age 32)

And others mentioned that:

‘Then they [*family*] gave advice the way they could, even now my little sister still calls to tell me that I should keep drinking treatment and not stop. The most important thing is to be grateful for life, you understand. They do give me courage and support.’ (P15, F, age 27)

And she went further to state:

‘Then they advise me that if I am feeling sick, I should do this, I feel like this I should do this. And that after drinking medication, I should drink more water as my medication needs me to drink more water. That I should drink water now and again so that I can be well hydrated […]’ (P15, F, age 27)

The motivation from family and friends to stay alive and keep on going regardless of the condition was also expressed by participants. Participant 13 emphasised:

‘[*T*]hey just told me to drink medication and I see that if I don’t, I will be killing myself and I have a young child with me. So, who will I leave him with? Even if I can be in denial, I know that I have the illness so I should just drink.’ (P13, F, age 30)

#### Category 1.2: Financial support

The participants explained how their families and friends help them financially as they are unemployed. Sometimes, it was very hard to get money for transport. Family members and friends support them in such circumstances. For example, a participant mentioned:

‘[*W*]hen I come to the hospital my sisters give me money for transport, and if my friend is not busy, she will accompany me here but just that today she was busy, so I came alone […]’ (P18, F, age 31)

Other participants also expressed that:

‘My mom said she will support me financially I shouldn’t be afraid to tell her […] my mom gives me the taxi fare, and if my little sister is at home, I can walk and come to collect medication.’ (P9, F, age 33)

Support from close friends was also seen as useful to help participants adhere to antiretrovirals (ARVs). Participant 18 added:

‘My friend helps me with money to come to the hospital because I’m taking a taxi to come here sometimes she gives me R20 to come to the hospital and I tell her that on my 15 days on this date and this date and then I don’t have the money she’s going to give me money.’ (P18, F, age 31)

### Theme 2: Facility support

Support from the attending facility was also seen as a supporting need that facilitated adherence to ARV. Support was in the form of regular counselling sessions and education on the treatment regimen.

#### Category 2.1: Counselling sessions

The counselling sessions that took place at the HIV clinics were described by participants, which indicates that facility support promotes ART adherence. Having a counsellor and a psychologist at the facility for instance was mentioned:

‘It’s the counsellor […] She changed my life, by saying that since I am positive and I should start treatment, and she explained about the treatment. She said I must learn to accept and that I shouldn’t think too much.’ (P4, F, age 28)

The counselling sessions provided at the health facility were described as useful and important during the initiation of ART. It helped participants in their overwhelming moment and assisted them in accepting the situation and understood the need to continue taking medication. This was verbalised by the participants:

‘But the support I get is that whenever I feel overwhelmed, I can come to the hospital and talk to the counsellors anytime.’ (P14, F, age 24)

‘It [*counselling*] helped me to accept earlier that I have this thing.’ (P17, M, age 31)

#### Category 2.2: Education on treatment

Education on treatment also emerged as a category of under facility support. Aside from the Government making the ARV medications available at all times, participants also highlighted the importance of consistent education in treatment. This sub-theme is expressed in the following quotes:

‘The day I started drinking them they explained that I should take them medication and not change time or skip times […] they said they make like […] like […] if you skip the germs become more powerful to work so I said then I won’t stop taking them.’ (P2, M, age 32)

‘So, at the clinic, they told me that, that when the treatment does this is because everything has a beginning and it will get used to my body, yes […] So, I continued taking medication, I stopped vomiting and feeling dizzy, so I saw that I didn’t have a problem.’ (P3, F, age 22)

‘They explained how the medication worked, that every month I need to come and collect medication then they gave me a card, and they said I should come even if I still have medication. They said I need to keep coming back so that they can check me.’ (P8, M, age 32)

Other participants also expressed how supportive the nurses were at the health facility. The frequent education and timing for taking medication among others are seen in the following quotes:

‘The people here are very supportive, and they will educate you so that you understand, they don’t judge or look down on people. They support us, and they teach you about this illness and the consequences of not taking treatment. They are very supportive […]’ (P5, M, age 27)

### Theme 3: Status disclosure

The category ‘Duration and disclosure to family and friends’ was explained by participants as the person they first disclosed their HIV status to and the period it took for them to disclose their status.

#### Category 3.1: Duration and disclosure to family and friends

Some participants preferred to disclose their HIV status to their close relatives. Parents and siblings were among those to whom participants disclosed their status. This is supported by the following quotes:

‘She [*mother*] was the first person. Then I told my father after I told them I called all my siblings, my sisters, and my younger sister since they are in Gauteng.’ (P9, F, age 33)

‘My brother is aware.’ (P2, M, age 32)

‘Umm at the first time I told my sister.’ (P18, F, age 31)

Others also preferred to disclose to their spouses, close friends and members of the extended family. As indicated in the following quotes:

‘I told my friend whom I spend most of my time with.’ (P10, F, age 26)

‘I told my aunt.’ (P6, F, age 31)

‘I told my husband, and he didn’t have a problem.’ (P19, F, age 26)

‘I told my brother’s wife she is a pharmacist and she also encouraged me to take medication.’ (P12, F, age 30)

‘It’s the counsellor, then my boyfriend.’ (P4, F, age 28)

Among the participants who disclosed their HIV status to close relatives, spouses and friends, the period was reported as just after confirming positive to after 3 months. As seen in the following participants’ quotes:

‘Aah […] the day I tested, when I left here, I told her because it had scared me, I was surprised, asking myself a lot of questions without answers.’ (P11, F, age 33)

‘Immediately after I got home from testing, I told them.’ (P15, F, age 27)

‘It was after 3 months.’ (P8, M, age 32)

Some participants also expressed that they were not ready to disclose their status and therefore, kept it a secret. This was because of personal disbelief, trust, family and friends’ reactions and fear of rejection. This is seen in the following quotes:

‘I didn’t tell anyone, it’s my secret. I don’t want to tell someone, then the next thing it is all over. Even my family will talk too much. I don’t want to tell them.’ (P1, F, age 29)

‘Like I was […] I was not ready to tell someone. I didn’t believe that I had this illness, I was not ready to tell someone that I was positive […] to be honest, I am a very secretive person and I think my friends are not as secretive. You understand, because if I told you today, the day you are angry with me you are going to use it against me and that will be the time where it will hurt me. That’s why I don’t want to.’ (P8, M, age 32)

Other participants expressed the fear of rejection, being sworn at, fights and being uncomfortable as reasons for not disclosing their status or disclosing after several months. The following quote supports this expression:

‘Those who were my friends and then they were HIV positive, and they felt rejected. They were rejected by their families and their friends so on and so on. So, I saw that this is how they treat people who are sick, no problem then I kept quiet.’ (P1, F, age 27)

## Discussion

The objective of this study was to explore the support needs of young adults initiated on ART. It was evident that there were support needs among these groups and when met they adhered to treatment. This information is critical in understanding the support needs, highlighting gaps in practice and tailoring interventions to support young adults. Participants mentioned that support from family and friends, support from attending facility and knowing whom you can trust enough to disclose your HIV status was essential when initiated on ART.

Social support from family and friends through motivation and encouragement plays a major role in ensuring that people living with HIV remains in care, as they become their primary source of support. In this study, the participants indicated that their family members and friends encouraged them, which was also found in Noroozi et al. (2017:7). Their family and friends were able to give health advice where they could, which indicates the importance of involving the family in the care of people living with HIV by educating them when they accompany their loved ones for clinic visits (Pius et al. 2021:6).

The participants felt inspired to continue taking medication knowing that they were not alone and had a family who cares for them. This statement is supported by studies conducted by Gugsa et al. (2017:12) and Martawinarti, Nursalam and Wahyudi (2020:154) who showed that patients who have support are more likely to continue taking treatment, as it increases the quality of life and their well-being.

In this study, 65% of young adults were unemployed, which made it difficult financially especially when they needed transport money to go for follow-up care. In a study carried out by Adeniyi et al. (2018:9), it was found that lack of money was a reason for non-adherence as people living with HIV feel that the side effects worsen when taken on an empty stomach because of lack of food. This was evident in this study whereby participants had to inform HCWs if they were unemployed or had no one to support them so that they could obtain a grant, until such a time that their health status showed improvement. Other participants depended on their family or friends for financial support to enable them to visit their clinic and therefore their socioeconomic status should not be neglected during the initiation of ART.

The participants indicated that they received counselling from HCW, which helped them continue taking medication, this was also evident in a study by Pius et al. (2021:6) who indicated that counselling impacts behaviour. A good patient and HCW relationship is essential as it enables people living with HIV to be open and share their struggles with HCW (Leyva-Moral et al. 2021:8). Counselling increases one’s knowledge about HIV and ART, increasing adherence (Molla et al. 2018:7), as it emphasises the benefits of ART, the importance of ART adherence and the goal of the treatment (Mabunda et al. 2019:4). Therefore, counselling decreases the barriers to care as coping strategies are given.

Proper education on medication can be a deciding factor for one to initiate or not initiate treatment. For the participants in this study, it was clear that they had enough information to decide whether to commence with medication or not. Education enables people living with HIV to make proper lifestyle changes (Scott et al. 2021:183). When education is not provided considering the people living with HIV’s lifestyle, it becomes difficult for the person to integrate treatment into their daily lives (Myers et al. 2020:10). Sileo et al. (2019:6) found that in cases where lifestyle changes are not made people living with HIV tend to skip medication as they want to drink alcohol, and this happens if proper education is not provided. These authors (Myers et al. 2020:10; Sileo et al. 2019:6) further emphasised the importance of ensuring that people living with HIV understands the demands of being initiated on ART and how important consistency is. Providing education about medication should be done to empower the people living with HIV to make better decisions. This is supported by Lumbantoruan et al. (2018:12) who found that people living with HIV remained in care because of post-HIV counselling, which was understandable and allowed them time to think. The people living with HIV should not be made to feel that they do not have a choice as that will result in them defaulting.

Education on ART is vital in ensuring that people living with HIV understands how it works and its benefits. It is evident that people who start ART when they already have symptoms or not feeling well tend to stop taking medication once they start feeling better (Murray et al. 2009:5; Sanjobo, Frich & Fretheim 2008:142). Such incidences can be avoided if proper education is given on initiation and as part of ongoing care. Ongoing education needs to be given to gauge the people living with HIV’s understanding and readiness to start ART assessed to improve chances of adherence (Pell et al. 2019:40). The findings indicate that newly diagnosed people living with HIV need to be given time to come to terms with the new diagnosis and for education to be a continuous process.

The duration for status disclosure ranged from the time of confirmation to 3 months after testing positive. People living with HIV often struggle to disclose their status to their loved ones because of fear of being viewed as unfaithful (Bhatia et al. 2017:7). Other participants refused to disclose their status because of the perceived stigma, fear of judgement and rejection. Horter et al. (2017:5) reported that patients who failed to disclose their status struggled to adhere to ART. This was also supported by Kamaingi and Meng’anyi (2019:75) who reported that patients who failed to disclose their HIV status were more likely to suffer treatment interruptions.

Disclosure of status has been found to promote status acceptance and support engagement in care (Horter et al. 2017:56; Moomba & Van Wyk 2019:4). This notion is also evident in a recent study conducted in Swaziland by Horter et al. (2017:56). Some participants in this study disclosed to their families and friends, and they acknowledged that disclosing their HIV status enables easy access to social support, encouragement and psychological support. This is consistent with the findings of Moomba and Van Wyk (2019:5) who reported that participants had no problem with ART adherence because of support from family members and close acquaintances.

## Conclusion

The study highlighted the need for the family to be involved in the treatment and care, as it can be an encouraging factor of their loved ones to adhere to ART. Services available to support people living with HIV were known and utilised by participants, as they were able to reach out to HCW when they had financial challenges. The support of young adults by HCWs was appreciated by participants as it assisted them to stay in care, which is critical to ensure continuity of care. Counselling and education by HCWs were performed thoroughly to ensure that the young adults understood the implications of stopping the treatment. Other participants still had difficulties in disclosing their status because of fear of stigma, therefore being at risk of defaulting on ART. This study indicates the areas that should be given attention during the initiation of ART to ensure that young adults have support.

### Recommendations

Further studies need to be performed to investigate how the family can be involved in the care of their loved one living with HIV. More studies are needed to investigate how young adults who are HIV positive have learnt to integrate their ART medication into their daily lives to avoid being lost to follow-up.

### Study’s limitations

Because of the sensitive nature of the topic being studied, participants may have been unable to disclose some of the important, yet sensitive, information.
